# Hormones and Psychosis: The Role of Estrogen in Schizophrenia

**DOI:** 10.1192/j.eurpsy.2024.386

**Published:** 2024-08-27

**Authors:** S. Jesus Magueta, A. L. Costa, G. Simões, A. I. Gomes, C. Madaíl Grego, P. Garrido

**Affiliations:** Departamento de Psiquiatria e Saúde Mental, Centro Hospitalar do Baixo Vouga, Aveiro, Portugal

## Abstract

**Introduction:**

Schizophrenia is a complex psychiatric disorder in which biological sex differences, have been extensively documented and researched. What is less well described, is what motivates these differences. Of the various proposed and explored reasons, estrogen appears to be one that has maintained some interest and promise. An increase in symptoms of schizophrenia has been observed to correspond with decreasing levels of estrogen in menopausal women, this, allied to the later symptom onset, culminated in the interest in this hormone and its role in psychotic illness.

**Objectives:**

The authors aim to briefly explore the current evidence on the association between estrogen and schizophrenia. Its relevance in symptom onset, protective status and eventual therapeutic applications will also be discussed.

**Methods:**

The authors conducted a brief non-structured narrative literature review using articles published in the Medline/Pubmed, ScienceDirect and Google Scholar databases. The keywords used during the research, alone or in combination, included: sex hormones, estrogen, schizophrenia and psychiatry.The studies consulted in this work included: cross-sectional studies, cohort studies, literature reviews and clinical case reports.

**Results:**

The literature exploring the relationship between the sex hormone, estrogen, and schizophrenia is extensive. Various studies confirm that during periods of estrogen withdrawal, women appear more susceptible to psychotic episodes. Results also demonstred that those with low estrogen, respond poorly to anti-psychotic drugs, whereas estrogen increased the efficiency of antipsychotics. In regards to symptoms, estrogen has been demonstrated to reduce the positive and cognitive symptoms of schizophrenia in the short term, thus being proposed as an eventual complementary treatment in those suffering from the disorder. It is known that estrogen regulates important pathophysiological pathways in schizophrenia, including dopamine activity, mitochondrial function, and the stress system.One of the explanations for this beneficial effect has been proposed to be action on cerebral blood flow and glucoce metabilism, as well as sensitizing postsynaptic dopamine receptors, thus serving as a protective agent against schizophrenia.

**Conclusions:**

The research appears to be pointing in the direction that estrogen appears to have an effect on psychosis in women, serving as a protective factor in these conditions as well as playing a significant part of the pathophysiology in schizophrenia. This influence on the pathophysiology, promises clinical pertinence, not only in a possible application so to attenuate positive and cognitive symptoms but also as a method to influence antipsychotic efficacy. Continued study in regards to the effects of sex hormones on the psychotic disorders is merited so as to further expand the tools in the mental health professional’s repertoire in the treatment of these serious mental illnesses.

**Disclosure of Interest:**

None Declared

